# Increased interleukin-10 and interferon-γ levels in *Plasmodium vivax *malaria suggest a reciprocal regulation which is not altered by IL-10 gene promoter polymorphism

**DOI:** 10.1186/1475-2875-10-264

**Published:** 2011-09-14

**Authors:** Tiago S Medina, Sheyla PT Costa, Maria D Oliveira, Ana M Ventura, José M Souza, Tassia F Gomes, Antonio CR Vallinoto, Marinete M Póvoa, João S Silva, Maristela G Cunha

**Affiliations:** 1 Laboratório de Microbiologia e Imunologia, Instituto de Ciências Biológicas, Universidade Federal do Pará, Belém, Pará, CEP 66075-970, Brazil; 2 Programa de Ensaios Clínicos em Malária, Instituto Evandro Chagas/SVS/MS, Ananindeua, Pará, CEP 67030-000, Brazil; 3 Laboratório de Virologia, Instituto de Ciências Biológicas, Universidade Federal do Pará, Belém, Pará, CEP 66075-970, Brazil; 4 Seção de Parasitologia, Instituto Evandro Chagas/SVS/MS, Ananindeua, Pará, CEP 67030-000, Brazil; 5 Departamento de Bioquímica e Imunologia, Faculdade de Medicina de Ribeirão Preto da Universidade de São Paulo, Ribeirão Preto, São Paulo, CEP 14040-030, Brazil

## Abstract

**Background:**

In human malaria, the naturally-acquired immune response can result in either the elimination of the parasite or a persistent response mediated by cytokines that leads to immunopathology. The cytokines are responsible for all the symptoms, pathological alterations and the outcome of the infection depends on the reciprocal regulation of the pro and anti-inflammatory cytokines. IL-10 and IFN-gamma are able to mediate this process and their production can be affected by single nucleotide polymorphisms (SNPs) on gene of these cytokines. In this study, the relationship between cytokine IL-10/IFN-gamma levels, parasitaemia, and their gene polymorphisms was examined and the participation of pro-inflammatory and regulatory balance during a natural immune response in *Plasmodium vivax*-infected individuals was observed.

**Methods:**

The serum levels of the cytokines IL-4, IL-12, IFN-gamma and IL-10 from 132 patients were evaluated by indirect enzyme-linked immunosorbent assays (ELISA). The polymorphism at position +874 of the IFN-gamma gene was identified by allele-specific polymerase chain reaction (ASO-PCR) method, and the polymorphism at position -1082 of the IL-10 gene was analysed by PCR-RFLP (PCR-Restriction Fragment Length Polymorphism).

**Results:**

The levels of a pro- (IFN-gamma) and an anti-inflammatory cytokine (IL-10) were significantly higher in *P. vivax*-infected individuals as compared to healthy controls. The IFN-gamma levels in primoinfected patients were significantly higher than in patients who had suffered only one and more than one previous episode. The mutant alleles of both IFN-gamma and IL-10 genes were more frequent than the wild allele. In the case of the IFNG+874 polymorphism (IFN-gamma) the frequencies of the mutant (A) and wild (T) alleles were 70.13% and 29.87%, respectively. Similar frequencies were recorded in IL-10-1082, with the mutant (A) allele returning a frequency of 70.78%, and the wild (G) allele a frequency of 29.22%. The frequencies of the alleles associated with reduced production of both IFN-gamma and IL-10 were high, but this effect was only observed in the production of IFN-gamma.

**Conclusions:**

This study has shown evidence of reciprocal regulation of the levels of IL-10 and IFN-gamma cytokines in *P. vivax *malaria, which is not altered by the presence of polymorphism in the IL-10 gene.

## Background

In human malaria, the naturally-acquired immune response can result in either the elimination of the infectious agent or a persistent response mediated by cytokines that leads to immunopathology, with activated T cells and macrophages, although the mechanisms are not well understood. High levels of pro-inflammatory cytokines, such as tumor necrosis factor (TNF), interferon-γ (IFN-γ) and interleukin-6 (IL-6) have been associated with severe pathologies [1-5], whereas low levels of regulatory cytokines, such as TGF-β and IL-10, have been associated with acute malaria [6-9]. Many aspects of this process remain to be understood, including the effects of cytokines on the control of the immune response and the differences between the protective and pathological modulatory effects. The IL-10 cytokine has an important role as an immunoregulator of the infections caused by *Plasmodium*, by neutralizing the effects of the other cytokines produced by Th1 and CD8 cells, which are responsible for much of the immunopathology associated with the overproduction of IFN-γ [8,10].

Infection by *Plasmodium vivax *induces a specific immune response by stimulating the release of cytokines from T cells and other mononuclear cells, which might have an important role in the parasite clearance by activating effector immune mechanisms [4]. This production of cytokines is an important stimulus of the phagocytosis that enhances the clearance of parasitized erythrocytes, but the inflammatory cytokines also mediate the pathological alterations that determine the manifestation of symptoms [5,11-13].

In *P. vivax *malaria, cytokines are released after the schizonts rupture, and elevated levels of TNF have been detected being released in pulses in paroxysms of synchronous *P. vivax *malaria and appear to be involved in the pathogenesis of malarial fever [11,13,14]. Other cytokines have been detected in *P. vivax *malaria, including IL-1β, IL-2, IL-4, IL-6, IL-8, IL-10 and IL-12 [13]. More recently, the participation of regulatory T cells in infection by *P. vivax *was showed [15], suggesting that the balance between pro and anti-inflammatory cytokines is required to control the pathological alterations. Comparing asymptomatic individuals and cases described as severe malaria, a study performed in Brazil showed that the IFN-γ:IL-10 ratio was higher in severe cases, while IL-10 levels were elevated in asymptomatic individuals [5].

In *P. falciparum *malaria, several studies have described an association between severe infections and enhanced pro-inflammatory cytokine response, including TNF, IL-1β, IL-6, and IFN-γ [1-3,16]. These cytokines are responsible for all the symptoms, pathological alterations and the outcome of the infection depends on the reciprocal regulation of the pro and anti-inflammatory cytokines. However, the balance between pro and anti-inflammatory response can be favourable to protection mediated by cytokine as IFN-γ [17,18]. Although many studies have investigated the serum levels and involvement of cytokines in *P. falciparum *malaria, little is known of the pro-inflammatory and regulatory balance during a natural immune response in *P. vivax-*infected individuals.

The factors that affect the production of these immune response mediators include a number of single nucleotide polymorphisms (SNPs) in cytokine-encoding genes, which affect gene expression. In the IFN-γ gene, the polymorphism at position +874 is located within a putative NFκB binding site and impairs the production of IFN-γ [19]. An association has also been observed between the +874T/T and +874A/A genotypes and high and low IFN-γ production, respectively, in patients with tuberculosis [20-22]. The IL-10 cytokine has a key regulatory role and its encoding gene contains a SNP in the promoter region (-1082G/A) which results in a reduction in the production of IL-10 in *P. falciparum *malaria [23] and may contribute to the immunopathology of a number of infectious diseases as leprosy [24], *Chlamydia trachomatis *infection [25] and tuberculosis [21,26,27].

As no previous study of *P. vivax *malaria has reported on the frequency of these polymorphisms, the relationship between cytokine IL-10/IFN-γ levels, parasitaemia, and their gene polymorphisms were examined. This study has shown evidence of reciprocal regulation of the levels of IL-10 and IFN-γ cytokines in *P. vivax *malaria, which is not altered by the presence of polymorphism in the IL-10 gene.

## Methods

### Patients and healthy control

Blood samples for cytokine measure were collected from a total of 132 patients, who were diagnosed as having *P. vivax *infection following examination of their thick blood smear. After the collection, all patients were given the standard treatment with 1500 mg of chloroquine in three days; 600 mg, 450 mg and 450 mg, respectively, and 30 mg of primaquine on the day diagnosis was made and in subsequent six days. The subjects in this group had had few or no previous recorded malarial infections. The control group was composed of 86 healthy individuals. The age, sex, number of malaria previous episodes, and history of other infectious diseases of each participant were recorded using a standard questionnaire. The healthy controls were selected from local blood donors with no parasitaemia or history of malaria. Blood samples were collected at the Pará state blood bank. The mean age (± SD) was 32.07 ± 13.40 years (range 12-68 years) in the infected group and 29.99 ± 9.24 years (18-48 years) in the control. Written informed consent was obtained from all participants, and the study was approved by the Ethical Review Committee of the Evandro Chagas Institute and conducted between 2007 and 2008.

### Blood collection

Two blood samples were taken from each patient. A finger tip smear was taken for the parasitological diagnosis, and then approximately 5 ml of venous blood was collected for the analysis of cytokines. The blood was drawn aseptically into Vacutainer^® ^tubes (Becton Dickson and company, Franklin Lakes, NJ, USA) and centrifuged at 1,200 g for 10 minutes at room temperature. The serum was separated out and the samples were aliquoted and stored at -20°C until assayed.

### Parasitological diagnosis

Thick blood smears were stained using 5% Giemsa solution and examined for *Plasmodium *species by two microscopists. Parasite density per μl of blood was estimated by counting the number of parasites per 100 fields. The number of asexual *P. vivax *was counted and double-checked blindly by an expert microscopist.

### Cytokine assays

The serum levels of the cytokines IL-4, IL-12, IFN-γ and IL-10 were evaluated by indirect enzyme-linked immunosorbent assays (ELISA), using pairs of cytokine-specific monoclonal antibodies provided by the commercially available test (BD, Biosciences - Pharmingen, San Diego, CA, USA). All tests were performed according to the manufacturer's instructions. Each plate included a standard curve of recombinant human cytokine run in parallel with the samples, and the limit of detection were 2.0 *p*g/ml (IL-4 and IL-10), 3.9 *p*g/ml (IL-12) and 1.0 *p*g/ml (IFN-γ). All samples were measured in duplicate, and the mean of the two values of optical density was used for all analyses.

### DNA extraction

Genomic DNA was extracted from the peripheral blood leukocytes of subjects *P. vivax *infected using proteinase K digestion and the standard phenol-chloroform procedure [28]. The DNA samples were stored at -20°C until analysis.

### Genotyping of *IFNG+874 *and *IL10-1082 *genes polymorphisms

The polymorphism at position +874 of the IFN-γ gene (rs 2430561) was identified by allele-specific polymerase chain reaction (ASO-PCR) method [19]. The PCR was performed in a final volume of 30 μl containing 500 ng of the total extracted DNA, 0.2 μM of each dNTP, 5 pmol/μl of each specific primer, 2.0 mM MgCl_2_, 50 mM KCl, 10 mM Tris-HCl pH 8.3 and 1.0 U *taq *DNA polymerase. The primers sequences used were: IFN-γ (+874) CP: 5'-TCA ACA AAG CTG ATA CTC CA-3', IFN-γ (+874) T: 5'-TTC TTA CAA CAC AAA ATC AAA TCT -3', or IFN-γ (+874) A: 5'-TTC TTA CAA CAC AAA ATC AAA TCA-3', which amplify a segment of 262 bp. The amplification reaction was performed under the following conditions: initial denaturation at 95°C for 5 minutes, 30 cycles of 40 seconds at 94°C, 40 seconds at 56°C and a final extension of 5 minutes at 72°C. The amplified product was analysed by electrophoresis in 2% agarose gel containing 5 μl of ethidium bromide (10 mg/ml) using transillumination with a source of ultra-violet light.

The polymorphism at position -1082 of the IL-10 gene (rs 1800896) was analysed by PCR-RFLP (PCR-Restriction Fragment Length Polymorphism) [29]. PCR was performed in a final volume of 50 μl containing 100 ng of DNA template, 200 μM of each dNTP, 200 nmol/μl of each specific primer, 1.5 mM MgCl_2_, 50 mM KCl, 10 mM Tris-HCl pH 8.3 and 1.0 U *taq *DNA polymerase. The following primers sequences used were: IL-10 (-1082) forward 5'-TCT GAA GAA GTC CTG ATG TC -3' and reverse 5'- CTC TTA CCT ATC CCT ACT TCC -3'. The 190 bp amplified product was subsequently incubated for 4 h with M*nl*I restriction enzyme and visualized in 4% agarose gel. After digestion the allele A is identified when is observed two fragment on electrophoresis (125 and 65 bp), whereas the allele G is identified in the presence of three fragments (93, 65 and 32 bp).

### Statistical analysis

To compare the concentrations of cytokines between infected and control group we used Mann-Whitney's U and student's t test. The relationship between levels of cytokines and parasite density and the number of previous episodes of malaria was tested using an analysis of variance. The distribution of genotypes was assessed according to the Hardy-Weinberg equilibrium. All statistical analysis was carried out in the BioEstat programme [30] and p values of less than 0.05 were considered statistically significant.

## Results

### Serum levels of pro- (IFN-γ and IL-12) and anti-inflammatory cytokines (IL-10 and IL-4) in the *P. vivax *and control groups

The levels of a pro- (IFN-γ) and an anti-inflammatory cytokine (IL-10) were significantly higher in *P. vivax *infected individuals as compared to healthy controls (p < 0.001). The mean concentration of IFN-γ was 44.50 ± 75.05 *p*g/ml in patients with malaria, and 1.47 ± 8.43 *p*g/ml in the control group, while the means for IL-10 were 283.80 ± 216.71 *p*g/ml and 2.07 ± 4.31 *p*g/ml, respectively. Concentrations were highly variable in the malaria patients, ranging from 0.0 to 332.85 *p*g/ml for IFN-γ, and from 0.0 to 586.33 *p*g/ml in IL-10. The mean concentration of IL-10 was 6.38 times higher than that of IFN-γ. There was also a positive correlation, but relatively weak correlation between IL-10 and IFN-γ concentrations in the patients (r = 0.389, p < 0.001). For the other two cytokines, IL-12 (pro-inflammatory) and IL-4 (anti-inflammatory), concentrations were very low or undetectable in most subjects, from both groups (Table 1).

**Table 1:** Serum concentrations of IFN-γ, IL-10, IL-4 and IL-12 cytokines in *P.vivax*-infected patients and the control group

Cytokine	Serum cytokine levels (*p*g/ml) Mean ± SD		
	
	Patients^1^	Control group^2^	*P values *
**IFN-γ**	44.50 ± 75.05 (0.0-332.85)^3^	1.47 ± 8.43 (0.0-68.09)	p< 0.0001
**IL-10**	283.80 ± 216.71 (0.0-586.33)	2.07 ± 4.31 (0.0-27.51)	p< 0.0001
**IL-4**	0.32 ± 1.48 (0.0-9.75)	0.0 ± 0.0 (0.0-0.0)	
**IL-12**	3.43 ± 12.65 (0.0-99.65)	7.03 ± 14.83 (0.0-80.71)	

^1^IFN-γ: n = 132; IL-10: n = 131; IL-4: n = 81; IL-12: n = 78;

^2^IFN-γ: n = 86; IL-10: n = 77; IL-4: n = 78; IL-12: n = 77;

^3^Range of values.

### IL-10 concentration increases in patients with high parasite density

The concentrations of IL-10 and IFN-γ were highest in subjects with the highest parasite densities (> 10,000 parasites/μl) in comparison with those with lower densities (≤ 1,000 parasites/μl), although the difference was only significant for IL-10 (Table 2). Mean levels of this cytokine were significantly different when comparing the lower and higher ranges of parasitaemia (≤ 1,000 *vs*. 5,001-10,000 parasites/μl: p < 0.05 and ≤ 1,000 *vs*. > 10,000: p < 0.05). In case of IFN-γ, however, the differences were not significant (p = 0.5671). The correlation between cytokine levels and parasite densities were low in both cases, but slightly higher in IL-10 (r = 0.1379) in comparison with IFN-γ (r = 0.0737).

**Table 2:** Effects of IFN-γ and IL-10 cytokines on parasite density and levels in primary exposure to *P.vivax *malaria

	Serum cytokine concentration (ρg/ml) Mean ± SD according to:						
	
	Parasites/μl				Number of previous malaria episodes		
	
Cytokine	≤ 1000 (n = 30)	1001 - 5000 (n = 34)	5001 - 10000 (n = 38)	> 10000 (n = 19)	0 episode (n = 12)	1 episode (n = 32)	> 1 episode (n = 41)
**IFN-γ**	30.87 ± 71.26 (0.0-324.55)^1^	37.81 ± 69.95 (0.0-287.69)	49.08 ± 71.99 (0.0-332.85)	63.22 ± 77.67 (0.0-298.88)	81.84 ± 102.59 (0.0-287.69)	29.05 ± 58.84 (0.0-298.88)	21.41 ± 49.42 (0.0-281.70)
**IL-10**	172.68 ± 84.56 (0.0-573.11)	298.77 ± 222.40 (4.06-576.89)	324.34 ± 212.07 (0.75-586.33)	364.31 ± 13.57 (0.0-579.41)	326.47 ± 196.09 (65.28-570.59)	184.78 ± 189.08 (0.0-533.45)	265.96 ± 215.08 (0.0-570.59)

^1^Range of values.

### IFN-γ levels were higher in patients with primary exposure to *P. vivax *malaria than in those with previous exposition

Serum IFN-γ and IL-10 levels were also analysed according to the number of previous malaria episodes. In the patients group who had been exposed to only one malaria episode, IFN-γ and IL-10 levels were higher than in patients who had had at least one previous episode (Table 2). Serum IFN-γ levels in primoinfected patients (81.84 ± 102.59 *p*g/ml) were significantly higher (p < 0.05) than in patients who had suffered only one (29.05 ± 58.84 *p*g/ml) and more than one previous episode (21.41 ± 49.42 *p*g/ml). A similar pattern was recorded for IL-10, although the differences were not significant (p > 0.05).

### Allele and genotype frequencies obtained for the IFN-γ and IL-10 genes

The mutant alleles of both IFN-γ and IL-10 genes were more frequent than the wild allele. In the case of the IFNG+874 polymorphism (IFN-γ) the frequencies of the mutant (A) and wild (T) alleles were 70.13% and 29.87%, respectively (Table 3). The mutant homozygote (AA) was detected in 50.65% of patients, while the AT and TT genotypes were recorded in 38.96% and 10.39%, respectively. Similar frequencies were recorded in IL-10-1082 polymorphism, with the mutant (A) allele returning a frequency of 70.78%, and the wild (G) allele a frequency of 29.22%. The mutant homozygote (AA) was detected in 49.35% of patients, while the AG and GG genotypes were recorded in 42.86% and 7.79%, respectively.

**Table 3:** Allele and genotype frequencies of the IFNG+874 and IL10-1082 polymorphisms

Polymorphism	Frequency			
**IFN-γ (IFNG+874)**	**Allele**	**n (%)**	**Genotypes**	**n (%)**
	
	A	108 (70.13)	AA	39 (50.65)
	
	T	46 (29.87)	AT	30 (38.96)

	**Total**	154 (100.00)	TT	8 (10.39)

			**Total**	77 (100.00)
	
**IL-10 (IL10-1082)**	**Allele**	**n (%)**	**Genotypes**	**n (%)**
	
	A	109 (70.78)	AA	38 (49.35)
	
	G	45 (29.22)	AG	33 (42.86)
	
	**Total**	154 (100.00)	GG	6 (7.79)
	
			**Total**	77 (100.00)

### Polymorphism affected the production of IFN-γ, but not IL-10

The frequencies of the alleles associated with reduced production of both IFN-γ and IL-10 were high, but this effect was only observed in the production of IFN-γ (Table 4). In the case of the IFNG+874 polymorphism, the mean serum concentration of IFN-γ was significantly lower (p = 0.0186) in the group with the AA genotype (30.85 ± 54.22 *p*g/ml) in comparison with carriers of the wild T allele (76.60 ± 99.54 *p*g/ml). Despite this difference, no significant difference was found between genotypes in mean parasite density (p = 0.4348), indicating that the IFNG+874 polymorphism does not affect the parasitaemia (Table 4). In the case of the IL10-1082 polymorphism, mean serum IL-10 levels were slightly lower for the AA genotype (301.47 ± 213.30 *p*g/ml) in comparison with carriers of the G allele (360.84 ± 199.00 *p*g/ml), this difference was not significant, indicating that IL10-1082 polymorphism does not affect IL-10 production in *P. vivax *malaria.

**Table 4:** Effect of genotype on serum concentrations of IFN-γ and IL-10 cytokines and parasite density

Genotype (n)	Serum cytokine level (*p*g/ml) Mean ± SD	Parasites/μl Mean ± SD
**IFNG+874 AA (39)**	30.85 ± 54.22 (0.0-281.70)^1^	5126 ± 4779.98 (12-20000)

**IFNG +874 AT and TT (38)**	76.60 ± 99.54 (0.0-332.85)	5917.76 ± 7516.64 (25-40000)

***P***	0.0186	0.4348

**IL10-1082 AA (38)**	301.47 ± 213.30 (4.68-578.46)	5287.49 ± 7392.26 (12-40000)

**IL10-1082 AG and GG (39)**	360.84 ± 199.00 (37.11-586.33)	5760 ± 5076.04 (200-20000)

***P***	0.1064	0.833

^1^Range of values.

In order to confirm that the IFNG+874 and IL10-1082 polymorphisms had no influence in the mechanisms of control or elimination of parasites, the parasite densities in patients with mutant alleles for only one or both of the genes were compared (Table 5). No association between these genotypes and parasite density was observed.

**Table 5:** Effect of association of the polymorphisms IFNG +874 and IL10-1082 on the parasite density

Genotype	Parasites/μl Mean ± SD	*P*
**IFNG+874AA + IL10-1082 AA (n = 19)** **IFNG+874AT or TT + IL10-1082 AG or GG (n = 20)**	4840.0 ± 5160.46 (12-20000)^1^ 6060.0 ± 5664.14 (200-20000)	0.2436
**IFNG+874AT or TT + IL10-1082 AA(n = 17)** **IFNG+874AA + IL10-1082 AG or GG (n = 18)**	3745.59 ± 3862.58 (25-12000) 5427.78 ± 4472.11 (200-17500)	0.0880

^1^Range of values.

## Discussion

The levels of both the pro- (IFN-γ) and anti-inflammatory (IL-10) cytokines were significantly higher in *P. vivax*-infected patients compared with healthy controls. However, serum levels of IL-12 and IL-4 cytokines were not detected. The concentration of IL-10 was higher in the group with high parasite density. This indicates that IL-10 has an important regulatory role in malaria infection, controlling the intensity of the immune response, as has been described in malaria experimental model and human malaria and in a number of other infectious diseases [7-9,31].

The immunopathogy of malaria is dependent on both the *Plasmodium *species involved and the regulatory mechanisms dependent on CD4 T cells. Recent studies comparing asymptomatic and severe *P. vivax *malaria suggest that IL-10 may be important for promoting protective immunity [5]. However, the factors responsible for the activation and regulation mediated by IL-10, and the timing of these controls, are not well understood.

Increased levels of inflammatory cytokines have been observed in cases of severe malaria. In *P. vivax *malaria, the IFN-γ serum level and the IFN-γ:IL-10 ratio were higher in patients with increased disease severity [5]. In severe *P. falciparum *malaria, the levels of both IFN-γ and IL-6 and the IL-6:IL-10 ratio were increased [1]. In this study, we observed the production of both pro (IFN-γ) and anti-inflammatory (IL-10) cytokines during infection, but IL-10 production increased when the parasite density was elevated. This study has identified for the first time a polymorphism in IL-10 gene in *P. vivax *malaria patients. As IL-10 is an effective regulatory cytokine, it is important to confirm its production levels and association with high parasite densities, and in particular, that the polymorphism (IL10-1082) does not affect its expression. The overall magnitude of the production and the imbalances between pro- and anti-inflammatory cytokines have been proposed as potentially important determinants of the control or exacerbation of clinical manifestations [8].

The role of IFN-γ and IL-10 has been described in *P. falciparum *malaria [8,17,18,32,33]. In the case of *P. vivax *malaria, studies in Brazil [34], Turkey [13], South Korea [35,36], and India [37] have analysed the production of these two cytokines, but have not recorded the serum concentrations of IL-12 and IL-4 in the same group of patients.

The comparison among groups with different levels of clinical manifestations has shown that in cases of mild *P. vivax*, the balance was in favour of IL-10, and the serum levels were significantly different between the groups with < 1,000 parasites/μl and > 10,000 parasites/μl. However, there was only a weak correlation between IL-10 concentrations and the number of parasites in the peripheral blood. This result may reflect the fact that the number of parasites in the bloodstream at a given moment may not always to show the maximum immunogenic potential able to induces response immune in the host, because part of the parasite's antigens can be in tissues of the immune system and not only in the blood.

The balance between pro- and anti-inflammatory cytokines is consistent with the induction of the regulation of the immune response against *P. vivax *malaria and it has been confirmed by an increase in the number of Treg CD25^+ ^cells during infection [15] and detection of high IL-10 levels in asymptomatic *P. vivax *malaria [5], indicating that the control was effective without provoking an imbalance of pro- or anti-inflammatory cytokines.

An increase in the percentage of FOXP3^+ ^Treg cells, IL-10-secreting Type I Treg cells (Tr1) and IL-10 levels has been recorded during acute *P. vivax *infection. The role of regulatory T cells has been confirmed in vivo and in vitro after stimulation of PBMC with crude antigens extracted from *P. vivax*-infected red blood cells, and it has been postulated that IL-10 can lead to a state of immunosuppression in *P. vivax *malaria by modulating different populations of cells, including a decrease in the number or activation of DCs [38]. This study confirms that this control can be mediated by IL-10, and suggests that the number of parasites can modulate the expression of the cytokine genes. While this and other studies are starting to some shed light on the participation of IL-10 in *P. vivax *malaria, many aspects of the regulatory response are not yet fully understood.

The analysis showed no significant association between the polymorphisms in the genes codifying INF-γ and IL-10 and the control of the parasitaemia. Although IL-12, INF-γ and IL-10 have been shown to play a significant role in the adaptive immune response in patients with *P. vivax *malaria [13,34], the IL-10 and INF-γ were detected, but not IL-12. The serum concentrations of IL-10 were similar to those found in patients from Brazil [34], but much higher than the levels recorded in patients from Turkey [13]. The levels of IL-10 were also elevated in *P. vivax *patients with fever [36] or hepatic dysfunction [39]. These results indicate that the serum levels of this cytokine may vary under different clinical conditions of the patients.

As IL-10 may exert its suppressive effects on INF-γ by down-regulation, it has been postulated that this mechanism may occur in other cytokines, including IL-12. Regulation of IFN-γ by IL-10 has been shown in vitro by adding anti-IL-10 human antibodies to PBMC cultures obtained from umbilical cords, which were stimulated with infected erythrocytes [40]. However, additional studies performed with samples collected at different time points throughout the course of *P. vivax *infection and the analysis of other cytokines with important roles in the induction and regulation of immunity to the intracellular stages of the parasite will be required to better evaluate their inter-relationship.

Different levels of cytokines concentrations were observed in Brazilian patients when compared with cytokines detected in the serum of the patients from Turkey [13], in which IL-4 and IL-12 were recorded, but the concentration of IFN-γ was not presented. Another study performed in Brazil found increased concentrations of IFN-γ in patients infected with *P. vivax *or *P. falciparum*, but increased IL-10 was observed only in *P. vivax *patients. In this study was confirmed the participation of IL-10, as described in previous studies [13,36], and the lack of any effect of the polymorphism in IL-10 gene on the production of this cytokine suggests that the maintenance of IL-10 is essential to the immune response.

The serum levels of IFN-γ varied considerably among individuals, although mean concentrations were lower than those recorded in a previous study of *P. vivax *patients from the Brazilian Amazonian, state of Pará [34]. These differences may arise from a combination of factors, such as the kinetics of the production of this cytokine and the patient's condition, given that IFN-γ levels may increase during fever episodes [36]. The IFN-γ concentration in *P. vivax *patients from South Korea who not had no hepatic dysfunction was low, whereas levels were relatively high in patients with hepatic disorders [39]. With regards to IFN-γ, this is an important finding because this cytokine is associated with the cellular response, and few studies of the cellular immune response in *P. vivax *malaria are available.

The production of both IFN-γ [41-45] and IL-10 [43-45] have been shown in PBMC culture stimulated with recombinant proteins containing antigens from blood stages of *P. vivax*. These studies indicates that antigen from *P. vivax *activates Th1 pathway, however, as few studies were performed, and this polarization of the subpopulation of lymphocytes remains controversial, further investigation may confirm more clearly the participation either Th1 and/or Th2 pathway in the protective response against *P. vivax*.

The production of IFN-γ is probably one of the most physiologically relevant effects mediated by IL-12 in the presence of inflammatory or immune stimuli, but in this study IL-12 was not detected. Similar results have been obtained for IL-12 in severe *P. falciparum *malaria [46]. With regards to *P. vivax *malaria, studies have detected IL-12, but not IL-4 in the same samples [13,34] or low IL-4 levels in cases with both hepatic disorders and normal liver function, whereas levels of IL-12 were high [39].

The concentration of IL-12 appears to increase at the beginning of the immune response, as observed in the in vitro study, which demonstrated an increase in the IL-12p40 transcripts in the first 24 hours after stimulation of PBMC with *P. falciparum *antigens, and a subsequent decrease to the basal level in 48 hours [46]. So, one explanation of our results would be that the concentration of this cytokine was at the basal level when the samples were collected. Another possibility is that IL-12 is regulated by IL-10 during this phase of the immune response [47-49], so that the cytokine that mediates this regulation may be present at higher concentrations. This suggests that IL-10 exerts suppressive effects on IL-12 through down-regulation. For a more conclusive analysis of this phenomenon, additional studies of samples collected prospectively throughout the course of the *P. vivax *infection will be necessary.

The participation of IL-4 in the humoral immune response to malaria involves the production of class IgG1 and IgG3 antibodies, which are involved in the response against the antigens of parasite erythrocytic stages [41,44,45]. While this antibody response has been well-documented in other studies, IL-4 cytokine production was not detected in response to *P. vivax*, This may indicate that the humoral immune response to malaria can be sustained independently of the production of IL-4, or that the serum concentrations of this cytokine may be alternate considerably over the course of the infection. On the other hand, the association between low levels of IL-4 and high of IL-10 suggests that, in this phase of the disease, the participation of the regulatory cytokine can be more important than the response mediated by cytokines from Th2 cells. In *P. falciparum *malaria, reduced serum levels of IL-4 has been described, indicating the participation of IL-10 in the regulation of the response mediated by IL-4 in *P. falciparum *malaria [16].

The high serum concentrations of IFN-γ were not associated with any reduction in the parasitaemia, which indicates no effective control of infection, in contrast with the evidence for *P. falciparum *malaria [33]. This may occur as a form of protection for the host, by avoiding an exacerbated response mediated by INF-γ. In addition, the production would be also negatively regulated by IL-10, and the parasite density may increase when the pro-inflammatory response is regulated by IL-10. This conclusion is corroborated by another study, which had recorded a positive correlation between the levels of IL-10 and parasite density in *P. vivax *patients [13].

The production of IFN-γ was higher in patients suffering their first exposure to malaria, suggesting that the effector response may be more intense during the first infection. In the case of IL-10, previous exposure to the parasite did not affect this cytokine production. This may be at least partly explained by the inefficiency of the cloning of memory cells. In malaria, protective immunity is acquired gradually through the natural exposure of individuals who live in areas where the disease is endemic, but this immunity is rapidly lost when this exposure ceases, indicating that the immunological memory of malaria is short-term, and requires constant exposure to the parasite [50,51].

The polymorphisms at position +874 of the gene that codifies IFN-γ and at position -1082 in the IL-10 gene was also investigated in order to establish whether the mutant homozygote genotypes are associated with reduced productivity of these cytokines, as well as analysing the possible implications of these polymorphisms for the parasite density in *P. vivax *malaria. While no previous research has focused on these polymorphisms in *P. vivax *malaria, a recent study analysed polymorphisms in the promoter region of the gene that codifies for TNF, and showed that allele A at position -308 and allele C at position -1031 are associated with increased serum levels of this cytokine, although there was no apparent effect on susceptibility to *P. vivax *malaria [52].

The A and T alleles of the IFNG+874 polymorphism had frequencies of 0.70 and 0.30, respectively, while in the IL10-1082 polymorphism, the frequencies of the A and G alleles were 0.71 and 0.29. These frequencies were similar to those obtained in studies of other infectious [53] and non-infectious diseases [54,55]. A study of the IFNG+874 polymorphism in leishmaniasis patients [53] recorded frequencies of 0.63 and 0.37, respectively, for the A and T alleles in patients with the cutaneous form of the disease, and 0.67 and 0.33 in those with the mucosal form.

In the IFNG+874 polymorphism, the homozygote mutant AA genotype was the most common, followed by AT and TT, the frequencies were, 0.51, 0.39 and, 0.10, respectively. These frequencies are similar to those found in patients with leishmaniasis [53] and tuberculosis [21]. The patients with the AA genotype had reduced serum levels of IFN-γ, while carriers of the wild allele (AT and TT) had high levels of this cytokine. This situation is similar to that found in studies of the *Mycobacterium tuberculosis *antigen [20,21] and antigens of the *Leishmania braziliensis *promastigote [53], which recorded low, medium, and high levels of IFN-γ, respectively, in the AA, AT, and TT genotypes.

The homozygote mutant (AA) was also the most common genotype in the IL10-1082 polymorphism, with a frequency of 0.49, in comparison with 0.43 for AG, and 0.08 for GG. Similar genotype frequencies were recorded in patients with severe anemia caused by *P. falciparum *[23]. The carriers of the wild allele (AG and GG) presented serum IL-10 levels higher than those with the homozygote mutant (AA), although the difference was not significant. The IL10-1082 polymorphism also had little effect on IL-10 levels in culture cells stimulated by *M. tuberculosis *antigens [27]. This study shows that neither polymorphism nor any combination of genotypes was associated with differences in the elimination of the parasite, as described for *P. falciparum *patients [56]. Taken together, our results suggest that a negative regulation mediated by IL-10 and the polymorphism in IFN-γ gene (IFNG+874) provides a control mechanism in *P. vivax *malaria. As production of the regulatory cytokine IL-10 was not affected by the polymorphism in its gene, these preliminary results suggest that the cytokines which activate effector mechanisms may be more susceptible to alterations than that the regulatory cytokine. In general, the resolution of infection requires a coordinated response in which initial pro-inflammatory mechanisms clear the parasite and then are down-modulated by IL-10 before pathology occurs.

This study has provided a first analysis of the polymorphisms in IFN-γ and IL10 genes, and confirmed the regulatory role of IL-10 in *P. vivax *malaria, which is not affected by this polymorphism. Moreover, increased IL-10 and IFN-γ in *P. vivax *malaria suggest a reciprocal regulation. Further immunological and molecular epidemiological studies are necessary in order to better understand the immunomodulation in both immunopathology and protective immune response scenarios in *Plasmodium vivax *malaria, essential for to development vaccines as well as new immunotherapeutics targets.

## Competing interests

The authors declare that they have no competing interests.

## Authors' contributions

TSM, TFG and MGC were responsible for cytokines detection. TSM and ACRV were responsible for genotyping. SPTC, MDO, AMV and JMS were responsible for selection of the patients and collect of the samples. JSS, MMP and MGC designed the study and discussion of the results. MGC wrote the manuscript. All authors read and approved the final manuscript.
